# Artificially-reconstructed brain images with stroke lesions from non-imaging data: modeling in categorized patients based on lesion occurrence and sparsity

**DOI:** 10.1038/s41598-022-14249-z

**Published:** 2022-06-16

**Authors:** Stephanie Sutoko, Hirokazu Atsumori, Akiko Obata, Ayako Nishimura, Tsukasa Funane, Masashi Kiguchi, Akihiko Kandori, Koji Shimonaga, Seiji Hama, Toshio Tsuji

**Affiliations:** 1 Center for Exploratory Research, Research & Development Group, Hitachi. Ltd., Tokyo, Japan; 2 Department of Rehabilitation, Hibino Hospital, Hiroshima, Japan; 3 Department of Neurosurgery and Interventional Neuroradiology, Hiroshima City Asa Citizens Hospital, Hiroshima, Japan; 4 Department of Neurosurgery, Graduate School of Biomedical and Health Sciences, Hiroshima University, Hiroshima, Japan; 5 Graduate School of Advanced Science and Engineering, Hiroshima University, Hiroshima, Japan

**Keywords:** Neuroscience, Diseases, Health care

## Abstract

Brain imaging is necessary for understanding disease symptoms, including stroke. However, frequent imaging procedures encounter practical limitations. Estimating the brain information (e.g., lesions) without imaging sessions is beneficial for this scenario. Prospective estimating variables are non-imaging data collected from standard tests. Therefore, the current study aims to examine the variable feasibility for modelling lesion locations. Heterogeneous variables were employed in the multivariate logistic regression. Furthermore, patients were categorized (i.e., unsupervised clustering through *k*-means method) by the charasteristics of lesion occurrence (i.e., ratio between the lesioned and total regions) and sparsity (i.e., density measure of lesion occurrences across regions). Considering those charasteristics in models improved estimation performances. Lesions (116 regions in Automated Anatomical Labeling) were adequately predicted (sensitivity: 80.0–87.5% in median). We confirmed that the usability of models was extendable to different resolution levels in the brain region of interest (e.g., lobes, hemispheres). Patients’ charateristics (i.e., occurrence and sparsity) might also be explained by the non-imaging data as well. Advantages of the current approach can be experienced by any patients (i.e., with or without imaging sessions) in any clinical facilities (i.e., with or without imaging instrumentation).

## Introduction

Brain imaging becomes one of immediate procedures as stroke patients are admitted to hospitals^1,2^. The imaging measure is critical to distinctly disregard hemorrhagic causes, determine stroke stages (e.g., acute, subacute, chronic), detect acute ischemic changes, recognize ischemic penumbra, and identify stroke occlusion^3^. Especially for subacute stroke patients, the strategic imaging is necessary to observe vasogenic edema^3^, potentially lengthen the intervention window, and monitor intervention effects^4^. The proper selection of imaging modalities and sequential procedure is preferable to manage salvageable ischemia and treatment decisions^3,5–7^.

The prompt disability assessment requiring the responsiveness is unlikely done for severe patients. Therefore, the information from brain imaging, such as lesion locations, are frequently used to anticipate consequent impairments. Global cognitive impairments were observed in patients with lesions at the left angular gyrus and left basal ganglia^8^. Axial- (i.e., frontal-posterior)^9^ and lateral-dependent^10^ lesions may influence distinct cognitive impairments. The damages at the right hemisphere were associated with the reduced attentional maintenance^11^. Furthermore, lesion locations characterize sub-domain dysfunctions of sensory-motor processing^12^, swallow^13^, and memory^14^. Worse mental conditions were found in stroke patients with lesions at the left frontal lobe, the bilateral basal ganglia, and the right Rolandic operculum^15,16^. Besides cerebral lesions, lesions at the anterior part of cerebellum may contribute to the severe case of ataxia^17^.

Recovery prognosis has been evaluated based on the initial brain imaging. Lesions occurred at specific regions affected the recovery outcomes^18^. Its effects were varied in a temporal manner. For example, lesions at the primary motor cortex and the caudate nucleus brought a poor prognosis of long-term gait recovery compared to the lesioned cingulum that specifically influenced a shorter course (6 vs. 3 months)^19^. Furthermore, the recovery of sub speech domains (i.e., fluency, comprehension, naming, repetition) were associated with lesion locations^20^. Both the follow-up imaging and the assessment of improved or worsened conditions are essential to monitor the effectiveness of rehabilitation.

The issue of limited instrumentation unfortunately raises, and the practicability concerns should also be addressed. Patients with restless and/or claustrophobic conditions feel inconveniences as undergoing the imaging process (e.g., magnetic resonance imaging/MRI)^21^. The presence of mental and electronics implants is a major constraint. The injection of contrast agent is cautiously done for patients with renal failures. The follow-up imaging becomes difficult to be routinely performed; this session is suggested to be carried out for only selective patients^22^ with worsened impairments^23,24^ after any interventions. The imaging information does important; obtaining that information with less inconvenience and high productivity is urgently strived.

Understanding the brain condition is beneficial not only for estimating post-stroke impairments and prognoses but also for early screening stroke onsets. Late reports of onset (about 1 h)^25^ may limit the eligibility for reperfusion treatment^26–28^. This phenomenon is commonly caused by patients’ unawareness of dysfunctions and confusion; several devices measuring patients’ systemic signals, motor and cognitive functions (i.e., non-imaging data) evaluate the risk of stroke^29^ which may be translated to brain conditions. Furthermore, the autonomic nervous system on physiological responses is associated with perceptive-cognitive functions and emotions^30–33^. The link between heartbeat, somatosensory perception, and brain (parietal and posterior cingulate regions) has also been confirmed^34^. Therefore, we hypothesize that the non-imaging data representing patients’ mind-brain-body functions is feasibly applied for estimating lesions.

**Figure 1:**
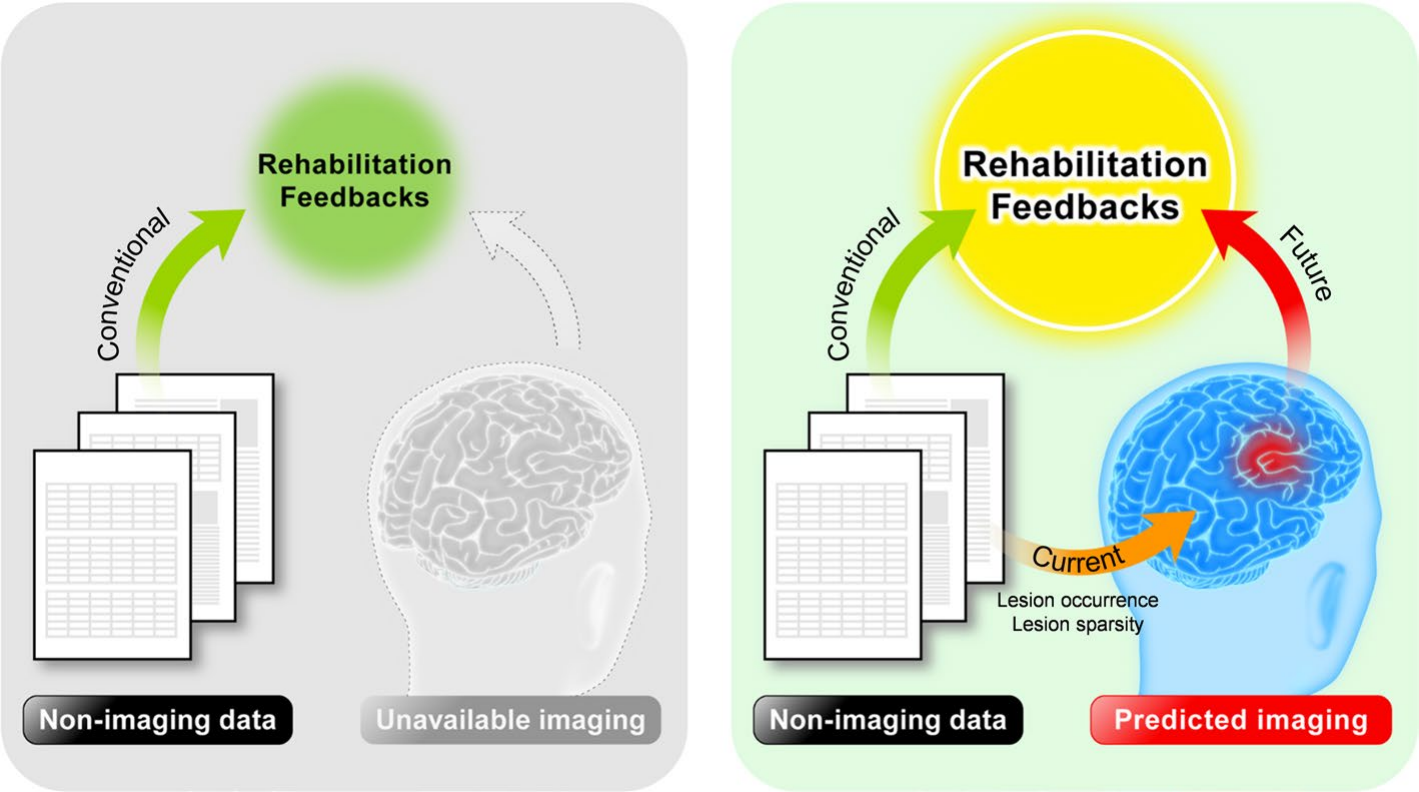
Concept of current study. In the conventional method, the rehabilitation feedbacks only rely on the assessed behavioral performance (i.e., non-imaging data) when the imaging measure is unavailable. In the current study, the non-imaging data is utilized to predict the brain imaging-like information. Lesion occurrence and sparsity are confirmed being influential factors in prediction models. The predicted imaging is purposely used for assisting rehabilitation feedbacks in the future as the environment is impractical for imaging sessions.

The current study aims to investigate the use of patients’ data and scores acquired from various standard tests as independent variables; those variables are fitted to the logistic regression model quantifying the probability of lesion occurrences for each brain region. Figure 1 depicts our concept on the current study. The evaluation metric comprises measures of accuracy, specificity, and sensitivity to examine performances of estimation models. The optimization process is also involved in selecting variables and employing analytical methods. The advanced technique, including artificial technology (AI), has been proposed to be implemented in the clinical field. The AI-aided detection of vessel occlusion was found to be cost-effective^35^. We are expecting that this lesion estimation can contribute to the advancement of healthcare providing more conveniences for patients, their families, and health professionals.

## Results

### Lesion occurrence and sparsity parameters for controlling lesion distributions

**Figure 2:**
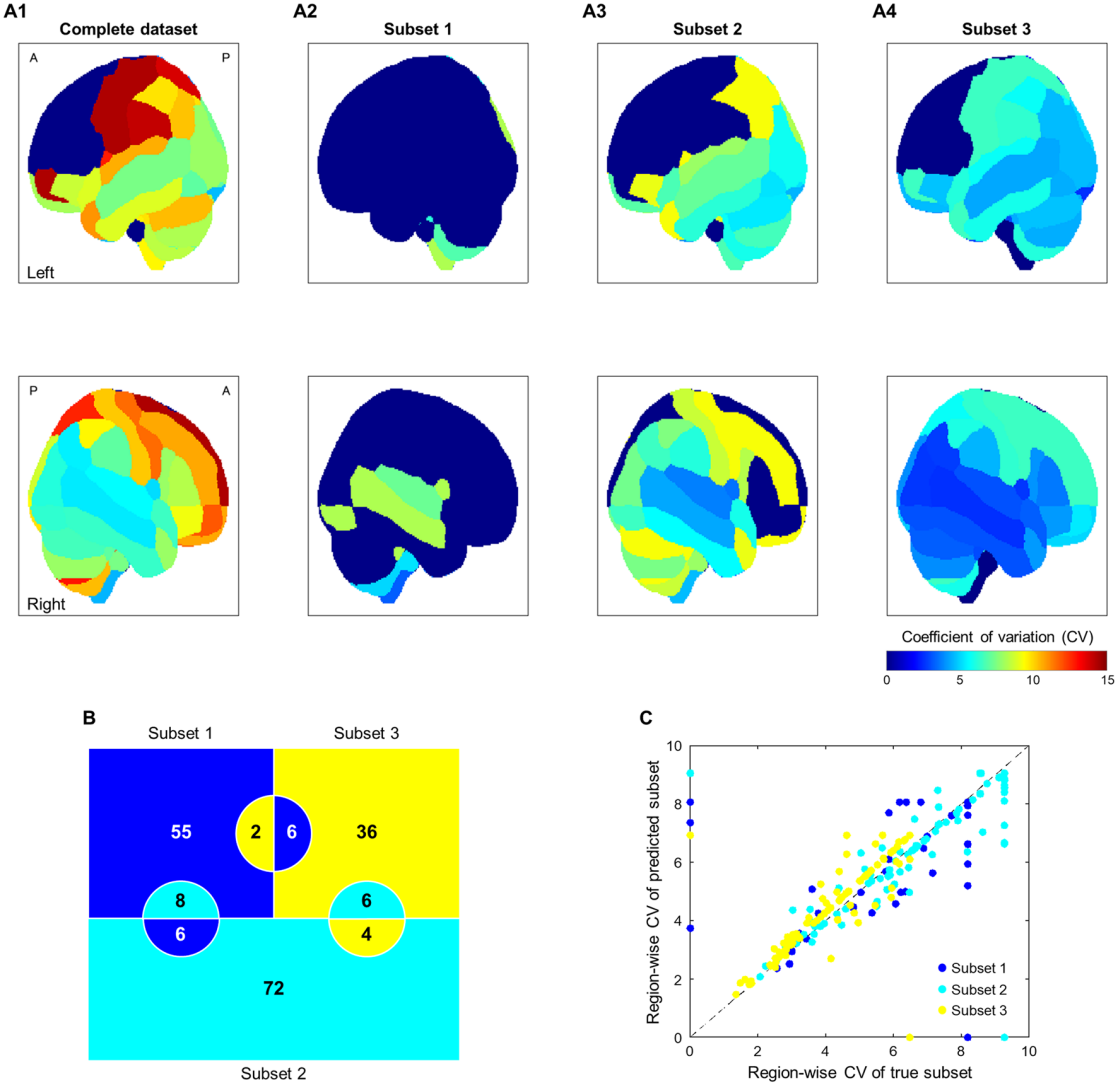
Region-wise lesion distributions and the performance of subject categorization. (**A**) Coefficients of variation (CVs) for lesion degrees across subjects in the complete dataset (A1) and three derived subsets (67-, 86-, and 42-subject subset; A2, A3, and A4 for subsets 1, 2, and 3, respectively). CVs are displayed for each region. The value of 0 CV denotes no lesion found in the respected regions (e.g., parts of the left frontal lobe) across subjects. (**B**) Subject categorization into three subsets (blue, cyan, and yellow colors for subset 1, 2, and 3, respectively) using the modeling of non-imaging data. The mis categorization for a specific subset (e.g., subset 1) is illustrated by two semicircles within other subsets (blue-colored semicircles in cyan- and yellow-colored squares). (**C**) Region-wise CVs in the true (*x*-axis) and predicted (*y*-axis) subsets (blue, cyan, and yellow-colored scatters for subset 1, 2, and 3, respectively).

Lesions were monitored by magnetic resonance imaging (MRI) and diversely found across brains in the dataset (*n* = 195). Lesion locations were specified following the Automated Anatomical Labeling^36,37^ (AAL; 116 regions). Lesion degrees in a region were sparsely distributed (right skewed). Its degrees were concentrated in 0%; high coefficients of variation (CV) were consequently observed. The well-distributed dataset is required in an analysis. Therefore, an approach was attempted to control lesion distributions across subjects. The subject data were clustered into three subsets based on lesion parameters—occurrence and sparsity (density of lesion occurrences across regions). Those subsets were specified by low occurrence—low sparsity (subset 1; *n* = 67), low-to-moderate occurrence—moderate sparsity (subset 2; *n* = 86), and moderate-to-high occurrence—moderate sparsity (subset 3; *n* = 42). Figure 2A shows lesion distributions for each specific dataset. The great gap of CV across regions (2.2 and 14 for left thalamus and left precentral, respectively) was observed in the complete dataset. Meanwhile, the gap between maximum and minimum CVs in the subsets 1–3 could be maintained by 5.1–7.2. The lesion modeling was then independently executed for each subset; the model performances were expected to be better in each subset than those of the complete dataset.

### Non-imaging data to model lesion characteristics

In order to realize the system with less imaging requirement, the categorization of subject data into subsets should also be feasible using non-imaging data. Therefore, the prediction of subsets using non-imaging features was attempted. About 40% of non-imaging features showed significant subset effects (Kruskal–Wallis *H* test; *p* < 0.05; *H*_(2,192)_ = 6.0–20). Those features were transformed to principal components (PCs); the weights of 7 PCs accounted for 95% of total weight. By performing the random forest analysis, those PCs were modelled to classify subjects into three subsets. From the model, PC importance was observed to be different for classifying each subset. The accuracy performance (i.e., true subset 1, true subset 2, and true subset 3) was adequate (83.6%). However, the model quality was less moderate with a Matthews correlation coefficient (MCC) of 0.29. Figure 2B illustrates a confusion matrix for the ternary classification. The hit rate was similar for each subset (82.1– 85.7%). Among subsets, subset 3 slightly showed a higher classification mismatch (10 out of 42). The greater distribution of lesion occurrence (subsets 3 vs. 1 and 2; 0.03–0.32 vs. 0.01–0.12) and the overlapped characteristic of lesion sparsity (subsets 3 vs. 2; 0.31–0.50 vs. 0.18–0.39) may explain this phenomenon. The attributes of region-wise CVs between the true and predicted subsets were found to be highly correlated particularly for the subsets 2 and 3 (Spearman’s *ρ* = 0.70; *p* < 0.05; Fig. 2C). Afterwards, the lesion estimation was performed on the predicted subsets (65, 82, and 48 subjects for subsets 1, 2, and 3, respectively). The successful subset prediction using only non-imaging data suggested the practical use of this system for the new data without imaging.

### Improved estimation performances in subsets with controlled lesion distribution

**Figure 3:**
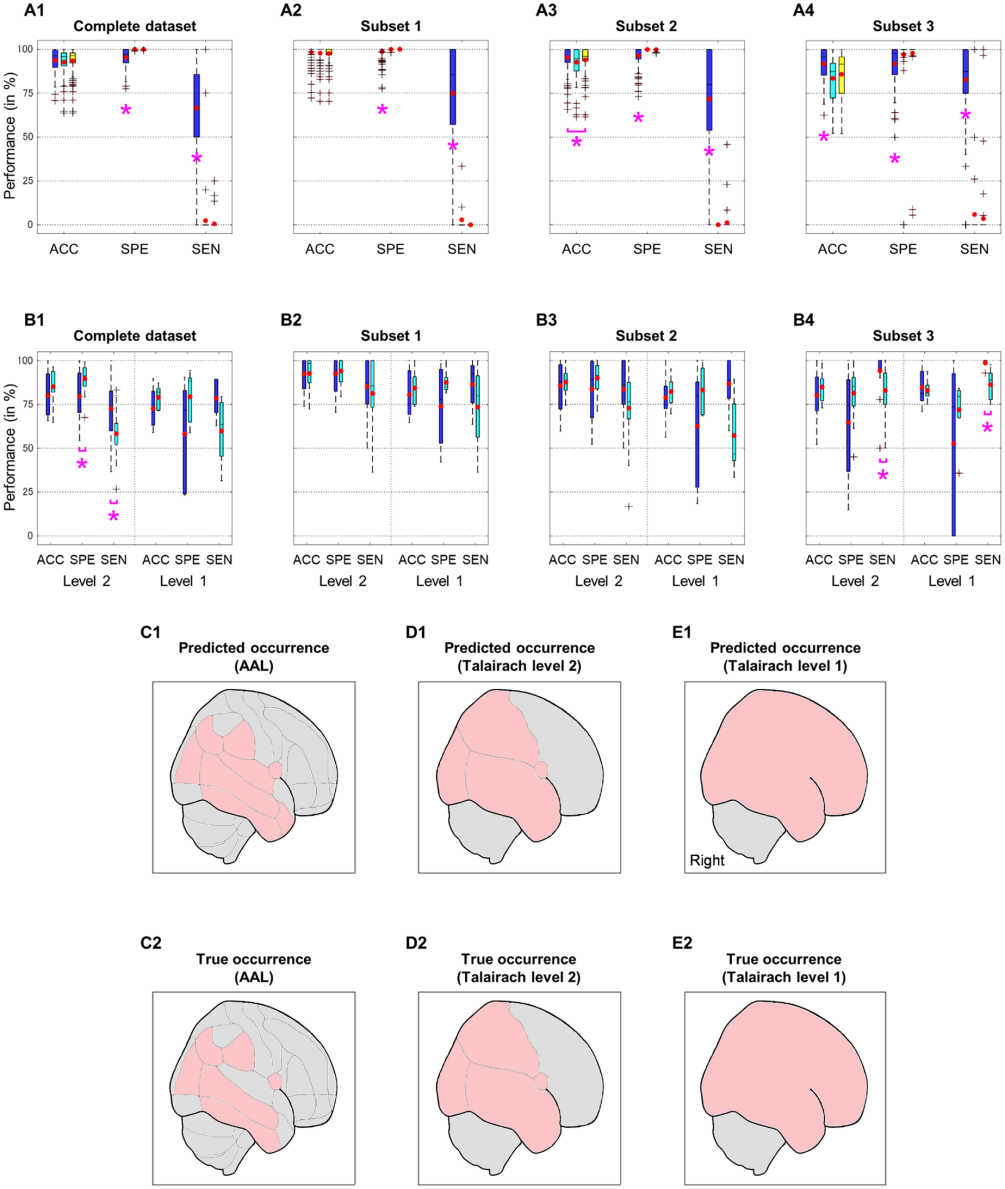
Results of lesion estimation. (**A**) Estimation performances [i.e., accuracy (ACC), specificity (SPE), and sensitivity (SEN)] across regions in the complete dataset (A1) and three derived subsets (A2, A3, and A4 for subsets 1, 2, and 3, respectively). The red-colored points represent the averaged performances across regions. The estimation was performed in three analysis manners—logistic regressions with a feature selection (blue-colored boxplots), L1 (LASSO; cyan-colored boxplots) and L2 (Ridge; yellow-colored boxplots). regularizations. The magenta asterisks show statistical significances (Kruskal–Wallis *H* test; Tukey–Kramer post hoc) among three analyses. (**B**) Estimation performances for the resolutions of Talairach levels 2 and 1 in the complete dataset (B1) and three derived subsets (B2, B3, and B4 for subsets 1, 2, and 3, respectively). The estimation processes were carried out by the spatially modified estimation (blue-colored boxplots) from a higher resolution (i.e., AAL) and by the direct estimation (cyan-colored boxplots) of respected resolutions. The performances of modified estimation were compared to those of direct estimation [Wilcoxon rank-sum test; *p* < 0.05(*)]. The red-colored points represent the averaged performances across regions of Talairach levels 2 and 1. An example (a 70-years old male) of subject-wise estimation in different resolutions of AAL (C1), Talairach level 2 (D1), and Talairach level 1 (E1). The red-colored regions denote regions with estimated lesions. These estimations are compared to the conditions of true occurrence in matched resolutions (C2, D2, and E2 for AAL, Talairach level 2, and Talairach level 1, respectively).

Instead of estimating lesion degrees, lesion occurrences [i.e., absence or presence (degree > 0%)] were predicted for each region (116 regions in total). Figure 3A shows boxplots of region-wise estimation performances [accuracy (true absence and true presence); specificity (true absence); sensitivity (true presence)] for each subset. The estimation was performed by logistic regression analyses; the effect of regularization on estimation performances was evaluated. Specificity and sensitivity performances were significantly influenced by the regularization process (Kruskal–Wallis *H* test; *p* < 0.05; *H* = 8.0–2.3 × 10^2^ ; *d*_*1*_ = 2; *d*_*2*_ = 79–313). Without the regularization process, the improved sensitivity was observed in all subsets (blue-colored boxplots in Fig. 3A; Tukey–Kramer post hoc). Meanwhile, by implementing the regularization, the specificity was found to be significantly higher (cyan- and yellow-colored boxplots for L1 and L2 regularizations, respectively) than that of resulted from the regression analysis without regularization. The accuracy also increased as the regression without regularization was conducted particularly in the subsets 2 and 3. As the specificity is optimized, the sensitivity consequently decreases, and vice versa. For a clinical screening application, the sensitivity performance was prioritized to minimize the negligence of lesion risks. Therefore, the regression analysis without regularization was selected as the optimum method.

The subset effect on accuracy was significant (Kruskal–Wallis *H* test; *p* < 0.01; *H* = 63–87 ; *d*_1_ = 3; *d*_2_ = 321–454) for any regression analyses (i.e., with or without regularization). The sensitivity of subsets was significantly greater than that of the complete dataset (subsets 2 and 3 > complete dataset, subset 1 ~ subset 2 ~ subset 3; Fig. 3: A3 and A4 vs. A1). Furthermore, the subsets 1 and 2 showed higher accuracy and specificity than the completed dataset and the subset 3 (Fig. 3: A2 and A3 vs. A1 and A4). According to these results, performing analysis on subsets with controlled lesion distributions obtained the better estimation; the importance of distribution control was confirmed.

### Applicable estimation models for any estimation resolutions

The term of spatial resolution hereby denotes how brain regions are specified. For the lower-level resolution, brain regions can be registered following the anatomical lobes (Talairach level 2; 18 regions) and hemispheres (Talairach level 1; five regions). On the other hand, the higher-level resolution with associated function labelling (e.g., AAL) provides more detailed regions. The estimation analysis was initially performed based on 116 AAL regions (see. “Non-imaging data to model lesion characteristics”). How the developed estimation models manage the modified resolutions was evaluated.

Figure 3B shows boxplots of region-wise estimation performances for resolutions of Talairach levels 2 and 1 in each subset. The estimation was carried out by two ways—(1) applying 116 AAL estimation models for the modified resolutions (i.e., modified estimation; blue-colored boxplots in Fig. 3B) and (2) developing estimation models using the corresponding resolutions (i.e., direct estimation; cyan-colored boxplots in Fig. 3B). The modified estimation brought a better (Wilcoxon rank-sum test; *p* < 0.05; *z* = 2.4–2.9) or equivalent sensitivity compared to the direct estimation. The slightly decreased specificity (Wilcoxon rank-sum test; *p* = 0.046; *z* = −2.0) in the modified estimation was shown in the complete dataset (Fig. 3B1; Talairach level 2). According to these results, the models of higher-level resolution (AAL) applicably or even more remarkably estimated lesions in lower-level resolutions. Newly developed models for lower-level resolutions are dispensable .

The effect of subsets on estimation performances was affected by the resolution. Its effect on the lesion estimation of Talairach level 1 was insignificant (Kruskal–Wallis *H* test; *p* > 0.05). However, the prominent subset effects were observed in both modified and direct estimations (Kruskal–Wallis *H* test; *p* < 0.05; *H* = 8.7–17 ; *d*_1_ = 3; *d*_2_ = 65–72) for predicting lesions of Talairach level 2. The complete dataset particularly demonstrated poorer estimation performances (Tukey–Kramer post hoc; subset 1 > complete dataset for accuracy and specificity; subset 3 > complete dataset for sensitivity) than other subsets did.

From the perspective of subject-wise estimation, the predicted occurrences were found to be similar to the true occurrences in all spatial resolutions as an example illustrated in Fig. 3C–E. Lesion at some regions of the right temporal and parietal lobes were seemingly overestimated. Those regions were located at the adjacent regions which the true occurrences were observed. As the spatial resolutions decreased, the sensitivity performances for the subject-wise estimation in all subsets significantly improved (Kruskal–Wallis *H* test; *p* < 0.05; *H* = 19–96 ; *d*_1_ = 2; *d*_2_ = 138–582).

The association between imaging and estimation results was further assessed. The Fisher’s exact test was performed on the contingency table (i.e., absence and presence for true and estimated conditions) regardless regions. The estimation results were plausible for any estimation resolutions and subsets (*p* < 0.05). The estimation procedure could properly mimic the results of lesion occurrence based on imaging. The insignificant association was found in several AAL regions (3–5 regions). Nevertheless, these insignificances were relatively minor (< 5%), the estimation models were worthwhile outcomes.

### Dominant estimation model inputs from cognitive- and motor-related tests

As the estimation analysis was independently performed, the region-wise models were acquired for each subset. The number of independent variables (i.e., inputs) was varied depending on regions. Cross-validation and re-sampling processes could not be performed for the limited number of region-wise lesions. Therefore, the risk of overfitting might be heightened for models of some AAL regions. The subset 3 required more variables (11 vs. 5–7 in median) for each region-wise model compared to the subsets 1 and 2 (Kruskal–Wallis *H* test; *p* < 0.01; *H*_*(2,223)*_ = 15; Tukey–Kramer post hoc).

Table 1 shows the frequency of standard tests used in models across regions. The frequency use was diverse between subsets; however, the most predominant tests were similar. Around (or more than) 50% of total modelled regions used non-imaging features acquired from the Behavioral Inattention Test (BIT), the Cognitive Assessment Tool (CAT), and the Mini-Mental State Examination (MMSE). The Functional Independence Measure (FIM) providing variables related to both motor and cognitive functions was also useful for estimation models especially in the subsets 1 and 3. Even though features of the Rivermead Behavioral Memory Test (RBMT) supported the estimation at more than 60% of total modelled regions for the subset 3, those features were unemployed for performing the estimation in the subsets 1 and 2.

**Table 1:** Frequency use of non-imaging features obtained from several tests as independent variables in estimating lesions for each subset.

Data and tests	Number of features	Frequency use across regions (in %)		
		Subset 1	Subset 2	Subset 3
Fundamental data	1	0	18.4	9.28
Behavioral inattention test	30	57.1	54.0	57.7
Cognitive assessment tool	65	47.6	75.9	79.4
Mini-mental state examination	23	50.0	63.2	58.8
Rivermead behavioral memory test	26	0	0	61.9
Trail making test	4	11.9	14.9	9.28
Bruunstrom recovery stage	3	0	0	10.3
Functional independence measure	23	50.0	12.6	34.0
Apathy scale	15	26.2	0	30.9
Hospital anxiety and depression scale	16	33.3	35.6	32.0
Japanese perceived stress scale	15	23.8	34.5	33.0
Medication information	7	19.1	27.6	32.0
Metabolite assessment	13	0	0	0
Total	241			

The mental-related tests, such as the Hospital Anxiety and Depression Scale (HADS) and the Japanese Perceived Stress Scale (JPSS), were moderately influential for about 20–30% of estimation models. Meanwhile, the variables from the metabolite assessment (e.g., blood) were inapplicable for any models. Some tests, including the Brunnstrom Recovery Stage (BRS) and the apathy scale, showed subset-specific uses. For example, BRS features were particularly involved in estimation models (e.g., right frontal–temporal-occipital, right cingulum, right insula, right thalamus, right heschl, and left cerebellum) for the subset 3.

## Discussion

The lesion information has been well employed to interpret post-stroke impairments and prognosis. In the current study, we attempted the opposite approach of lesion prediction that is rarely pursued to provide the lesion information related to impaired and intact functions. We confirmed the feasibility of non-imaging data (e.g., cognitive-motor functions, mental conditions, etc.) used for estimating the occurrence of lesioned brain regions. These results implied the bi-directional relationships between lesions and post-stroke functions.

The non-imaging features collected from several standard tests were used in estimation models for major regions. Those standard tests, including MMSE, FIM, a part of CAT (e.g., digit span), were commonly conducted to screen and/or evaluate deficits of global and higher-level cognitions^38^. In the practical framework, cognitive and mood assessments have been implemented using specifically different batteries for each clinical stage (e.g., pre-stroke, hyperacute, rehabilitation)^39^. Even though the validity of assessment tools are still argued^40,41^, the importance of neuropsychological assessment is well agreed. Cases of post-stroke dementia^42^ and psychiatric disorders (e.g., depression, apathy)^43–45^ became one of rationalizations for both cognitive and mood assessments. Post-stroke cognitive and neuropsychiatric impairments raised major concerns from all entities (e.g., stroke survivors, caregivers, and health professionals)^46^.

Widely located lesions were contributed to global cognitive deficits^47^. Those locations were associated with vascular trees rather than random incidents^8^. The hypothesis of vascular regulation on functional brain networks was proposed^48^. The lesion-evoked effects on vascular regulation and its causal impacts on brain network have not be investigated yet. However, the relationship between post-stroke connectivity disruptions at extensive brain networks (e.g., dorsal attention, auditory, sensorimotor, visual, etc.) and multidomain impairments (e.g., motor, memory, language, etc.) has been reported^49^. These disruptions may occur due to reduced interhemispheric connectivity within networks and increased intrahemispheric connectivity between networks^50^. Furthermore, a straightforward insight of disconnected connectomes caused by lesions had been suggested^51^.

In previous study, it was investigated that a sole mental domain, and the risk of worse mental conditions may be related to lesions at several regions^15,16^. The causal association of those lesion-specific locations is still unclear. The involvement of several networks (affective, cognitive control, default mode, and reward networks) modelled in a specific pattern tried to explain the symptomatic depression^52,53^. Depressive-related deficits on cognition have been frequently observed^54^; the engagement between impaired cognitive processes and emotional dysregulation is proposed^55^. High-degree brain hubs were found to be responsible for compounding cognitive functions^56^. The functional and structural segregation of each network toward a specific deficit domain is seemingly implausible, and vice versa. Therefore, the uses of features from multiple standard tests in estimation models of multiple brain regions are well-grounded. According to the results, some performed tests showed a little inclusion (or even none) in estimation models. The irrelevant tests can be reduced for the next study; the data collection period will be accordingly shortened.

We introduced a novel concept of sparsity measure. This measure quantifies the density of lesion occurrences. The lesion degree (i.e., ratio between lesioned and total voxels) was disregarded; the lesion location was implicitly comprehended. Subjects’ lesion sparsity and occurrence were characterized by using non-imaging features with high classification performances (83.6% accuracy). This result confirmed the practicability of non-imaging data to explain imaging data to some extent. One of foremost criteria for a good modeling is the sufficient sample number^57^. The dataset should be well distributed. However, the actual datasets have limited samples and often follow non-normal distributions^58^. The study with a small number offers a shorter research time^59^. Collecting more samples does require greater cost. Therefore, the small study is frequently attempted to provide a preliminary evaluation for hypotheses. The subject classification based on the lesion sparsity into some subsets brought a technical advantage. The subsets consisted of a smaller sample number. We tried our best to minimize the risk of overfitting by performing the processes of cross-validation and re-sampling. The better-performing models were consequently obtained from the subsets with reduced lesion variances. Therefore, the measures of lesion sparsity and occurrence presented relevant subject attributes.

More advanced machine learning methods (e.g., neural networks) have been extensively implemented for stroke studies^60^. Those methods had been applied on both imaging data and non-imaging data for multiple purposes (e.g., outcome prediction, risk assessment, onset identification)^61–63^. We currently tried two machine learning methods—random forest (ensemble model) and logistic regression for subset classification and lesion estimation, respectively. Each method performed well for its target. Some studies elaborated the comparison between classical (i.e., logistic regression) and advanced machine learning methods. Both classical and advanced methods showed equivalent performances^64,65^. However, the method suitability may depend on the dataset. Therefore, the suitability of machine learning methods will be advantageously evaluated for the next study. The trade-off between performance and computation costs should be considered as well.

For stroke cases, the prognostic outcome is the main concern. The theory of neuroplasticity has been introduced and studied. While the lesion volume is likely unchanged or even expanded^66^, the reorganization triggered by compensatory responses leads the recovery^67^. The recovery is progressive and time-dependent^68^. The utilization of estimation models is also envisioned during the recovery course. The reduced number of required imaging session is anticipated. The usefulness of estimation was improved by providing the subset classification based on only non-imaging data to select subset-specific models. Therefore, patients who inconveniently perform the follow-up imaging can receive similar benefits functionally addressing the neuroplasticity-evoked effects. The current lesion estimation is also purposeful for three different resolutions. Observed lesions at the lower spatial resolutions (i.e., greater region volume) predict wide-ranging impairments. The care feedback managing broader functions may consequently suggested. Those cares are expectedly optimized and personalized for each case. We hypothesize that the altered subset categorization (i.e., changed sparsity and total occurrence) and region-dependent occurrence during the rehabilitation course may be relevant to the network reorganization. Unfortunately, the longitudinal data, including both imaging (topology, functional) and non-imaging data, is unavailable for the moment. The verification of our hypothesis should be addressed in the near future.

Despite our achievements through the current study, we encountered four limitations. *First*, the low sample number entailed modeling disadvantages. Even though the modeling processes had been independently done for each subset, the model performances were region-dependent. The sample numbers for lesion classes (i.e., absence and presence) were found to be imbalanced (0.012–1.7 ratio between numbers of presence and absence across regions). The processes of cross-validation and resampling have been carried out; the risk of overfitting may unavoidably remain to some degree. As the feasibility of lesion estimation has been confirmed in this small dataset, the more accurate reliability of estimation models and reproducibility of estimation performances should be investigated using a greater dataset. *Second*, the estimation was only applicable to lesion occurrences not to lesion degrees (i.e., volume, size). The estimation of lesion degrees was unsuitably carried out in the current dataset with the right-skewed distribution of lesion degrees. More data acquisition with well-distributed lesion degrees for each region is highly requisite for modeling. Lesion degrees have been highly corresponded to functional impairments and prognostic recovery in both human and animal studies^69–71^. The lesion variables have been extended to its affected structural^72^-functional^73^ topology and activation^74^. In the future study, the estimation of lesion location and degree should consider clinical manifestations toward symptomatic impairments and restored functions. *Thrid*, the current analysis only involved T2W images as reference data. However, an image system analyzing both diffusion and perfusion images has been constructed. Thus our estimation method will be applicable to more diverse imaging methods in the future. *Forth*, some standard scales [e.g., National Institute of Health Stroke Scale (NIHSS), Fugl-Meyer Assessment (FMA)] for the severity and/or recovery stages of patients were not described. The NIHSS is commonly used to determine the severity of acute stroke patients; in this study, the patients were in rehabilitation stage after acute-phase treatment. Therefore, the assessment of activities in daily living using the FIM test was more appropriately conducted for evaluating patients’ severity. The FMA has been widely employed to monitor recoveries. However, its complex and lengthy procedure hindered the application in clinical practices. The inclusion of other standard tests (i.e., NIHSS FMA) in models will be an issue for future study.

In conclusion, we confirmed that non-imaging data feasibly predicted lesion occurrences at brain regions. The lesion occurrence density (i.e., sparsity) together with the lesion occurrence characterized stroke patients, and this sparsity measure was also associated with non-imaging data. The high-resolution imaging systems are not always available for all clinical facilities. The current approach is expected to deliver the merits of understanding lesion information even in the challenging environments. Even though further sample acquisition and analysis are required, the current approach may be prospectively feasible for acute and subacute stroke settings. The extension of model usages for specific acute (e.g., detection of ischemic penumbra and occlusion presences) and subacute (e.g., observation of corticospinal tract, Wallerian degeneration, brain atrophy, and connectivity alteration) cases will demonstrate advanced clinical benefits. The effects of employing the estimated lesion profile on rehabilitation outcomes will be assessed further by also comparing the results of conventional function monitoring.

## Methods

### Subject

Two hundred eight patients (170 males; 66.9 ± 9.8 years old; range 39–86 years old) with cerebral infarction participated in this study. A part of this dataset has been reported in the previous publication^16^. Patients were initially admitted to hospitals and then transferred to the rehabilitation ward of Hibino Hospital, Hiroshima. Severity conditions were widely distributed; the admission timing to the rehabilitation ward was consequently different (0–90 days after stroke onset) across patients. Patients were recruited during their rehabilitation periods. Among 208 patients, 42 patients suffered recurrent stroke. Thirteen subjects were excluded (12 males; 65.4 ± 9.9 years old; range 42–80 years old) due to limited lesions occurred in the brainstem. This study was approved by the ethics committee of the Shinaikai Hibino Hospital and the Hiroshima University Epidemiological Research. All experiments were conducted in compliance with relevant regulations and the latest version of the Declaration of Helsinki. Informed consent was obtained from either patients or those authorized to provide consents on behalf of patients, including family members or guardians, before we started any measurements.

### Imaging data

Patients’ brains were scanned using a 1.5-T MRI instrument to obtain T2-weighted images (GE Healthcare, Milwaukee, WI). Imaging data were examined to quantify the brain lesions. Brain scans were normalized in accordance with the brain template of Montreal Neurological Institute (2 × 2 × 2 mm^3^ voxel size). Lesions were visually assessed and manually labeled (MRIcron, https://people.cas.sc.edu/rorden/mricron/index.html) afterwards by a proficient doctor (KS). Binary masks (1 for lesioned voxels, and vice versa) were created for non-smoothed lesions. The process of lesion identification is described in detail elsewhere^11^. In order to understand region-dependent lesions, binary masks were spatially registered following the AAL^36,37^ into 116 regions. The ratio between lesioned and total voxels for each region was computed (in % unit). Those ratios were hereafter referred as the ”lesion degree measure”.

As mentioned above, severity conditions were varied shown in wide-range numbers of lesioned regions across patients. The region-wise distribution of lesion degree measure was also dispersed. Frontal lesions were unlikely observed in patients; more than one-fifth patients showed lesions in the hippocampus, thalamus, and temporal lobe. These dispersed distributions may interfere the modeling process of lesion estimation. Therefore, 195 patient data were categorized into smaller subsets for minimizing the lesion distribution dispersion.

**Figure 4:**
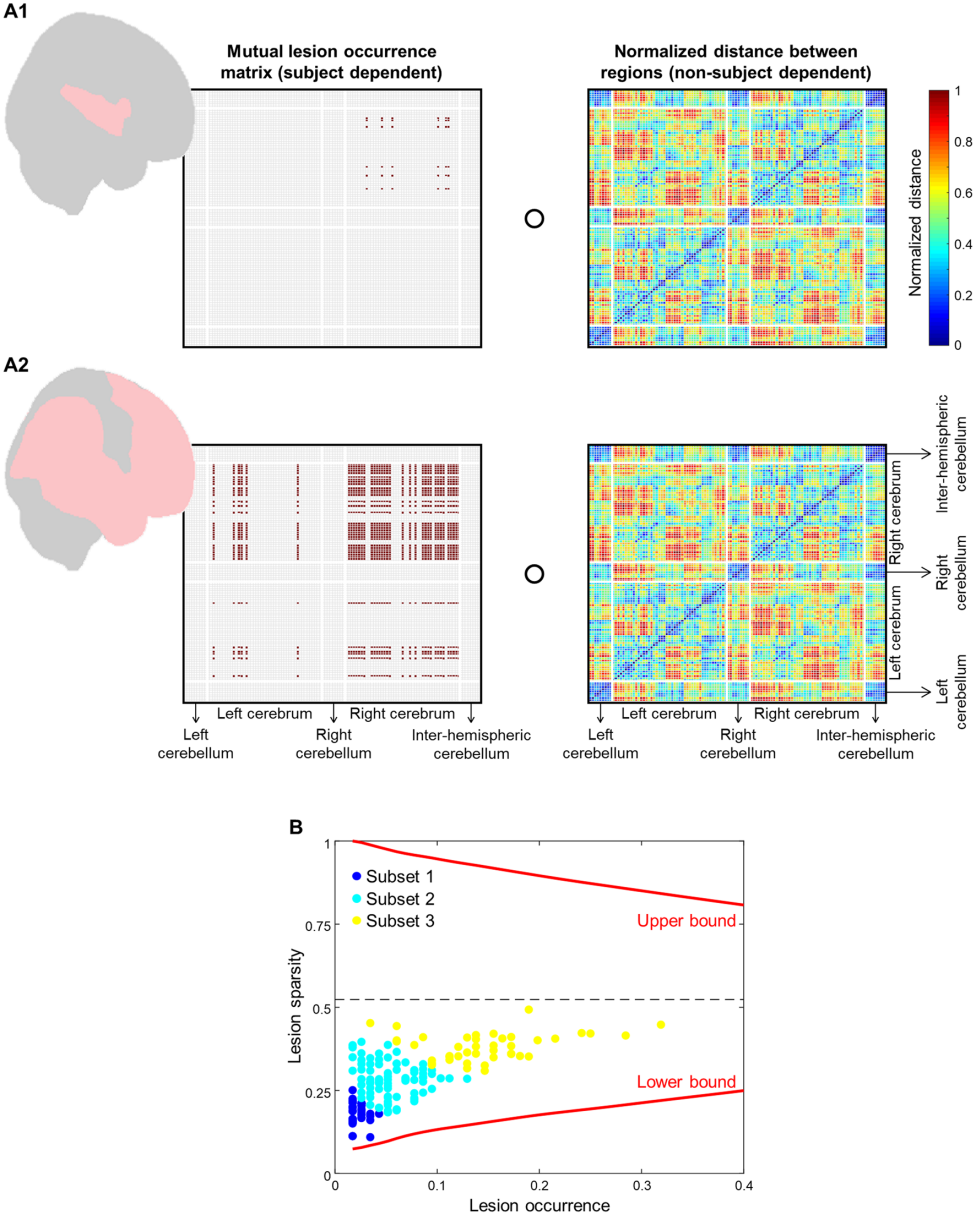
Subject categorization based on lesion characteristics. (**A**) Illustration of lesion sparsity computation [i.e., Hadamard product (“open circle”) of mutual lesion occurrence (left matrices; values of 1 or NaN) and normalized distance (right matrices; 0–1 value range)] from two subjects with low (A1) and high (A2) lesion occurrences. The red-colored regions on the brain model represent regions with lesions. (**B**) Scatter plot of lesion sparsity (*y*-axis) versus lesion occurrence (*x*-axis). Each point represents each subject data; blue-, cyan-, and yellow-colored points denote subject data in subsets 1 (67 subjects with low occurrence and low sparsity; 23 subjects have only one lesion location), 2 (86 subjects with low-to-moderate occurrence and moderate sparsity), and 3 (42 subjects with moderate-to-high occurrence and moderate lesion sparsity), respectively. Subject data are limited within upper and lower bounds (red lines).

Patient data were characterized by two parameters, named lesion occurrence and lesion sparsity. The parameter of lesion occurrence quantifies a ratio between the number of regions with lesions (degree > 0%) and the number of total regions (i.e., 116 regions). Meanwhile, the parameter of lesion sparsity indicates a density measure of lesion occurrences across regions. This measure is interpreted by the averaged Hadamard products of mutual lesion occurrence (1: AND logic, NaN: OR logic; see Eq. (1)) and normalized distance (i.e., average of voxel-to-voxel distances using the AAL template references) matrices between regions.

1 $$Mutual\, occurrence= \left[\begin{array}{l}if \,\,\,  {D}_{m} > 0\% \wedge {D}_{n}> 0\% \to 1 \\ else \to NaN\end{array}\right.$$

where *D* is lesion degree of region *m*, *n*, etc. Figure 4A shows examples of lesion sparsity computation from two subjects having low and high mutual occurrences, respectively. The greater magnitude of lesion sparsity denotes the lower lesion occurrence density between regions. Figure 4B visualizes the lesion sparsity versus lesion occurrence plot. *k*-means clustering (*k* = 3) was then performed on two parameters resulting in three smaller subsets. Subsets 1–3 were distinguished by low occurrence—low sparsity (67 samples), low-to-moderate occurrence—moderate sparsity (86 samples), and moderate-to-high occurrences—moderate sparsity (42 samples), respectively. We hypothesized that the estimation performance may improve as implementing a dataset having relatively uniform lesion conditions (e.g., occurrence, sparsity) on the modeling process.

### Non-imaging data

During the rehabilitation periods, patients’ cognitive and motor functions were assessed using standard batteries. Memory, attention, arithmetic, language, visual, spatial, and higher order functions were evaluated by performing the Cognitive Assessment Tool (CAT; digit/tapping span forward/backward tests, visual cancellation test, symbol digit modalities test, continuous performance test, position Stroop test, paced auditory serial addition test; auditory detection test, memory updating test), Mini-Mental State Examination (MMSE), Behavioral Inattention Test (BIT), Rivermead Behavioral Memory Test (RBMT), and Trail Making Test (TMT; Parts A and B). Functional disabilities and recovery progresses were estimated using the Functional Independence Measure (FIM) and the Brunnstrom Recovery Stage (BRS) at the times of admission to and discharge from the rehabilitation ward. Besides cognitive and motor functions, patients’ mental conditions were monitored using Japanese versions of the Apathy Scale, Hospital Anxiety and Depression Scale (HADS), and the Perceived Stress Scale (JPSS). Furthermore, the blood test was performed to measure levels of white/red blood cells, hemoglobin, etc. Demographic data (e.g., age) and medication information (e.g., doses) were also included as the non-imaging features. By excluding categorical variables and variables collected at the discharge time, 241 variables were used as potential inputs of lesion estimation models (see Table 1).

### Model for subset selection

The subset categorization was done based on the imaging data. Recovery courses may alter lesion conditions; its effects on occurrence and sparsity have not been investigated yet. To minimize measurement numbers, lesion occurrence and sparsity were favorably characterized by non-imaging data. Prior to the process of lesion estimation, non-imaging features were modelled to classify subjects into one of subsets (low occurrence—low sparsity, low-to-moderate occurrence—moderate sparsity, and moderate-to-high occurrences—moderate sparsity). The supervised multiclass classification was executed using the random forest in open-source PyCaret library (https://pycaret.org/about; Python programming language). Features with excessive missing data were eliminated. Subset effects on non-imaging features were evaluated (Kruskal–Wallis *H* test); features with significant subset effects were selected as potential independent variables. Afterwards, the feature dimensionality was reduced by performing the principal component analysis (95% of total weight). Cross-validation (75% training data, threefold) and re-sampling (100 times) were also conducted to obtain robust models. Hyperparameters were tuned to optimize MCC as a measure of model quality. An optimum model was selected based on the highest MCC respected to the highest probability of correct rate.

### Lesion modeling process

**Figure 5:**
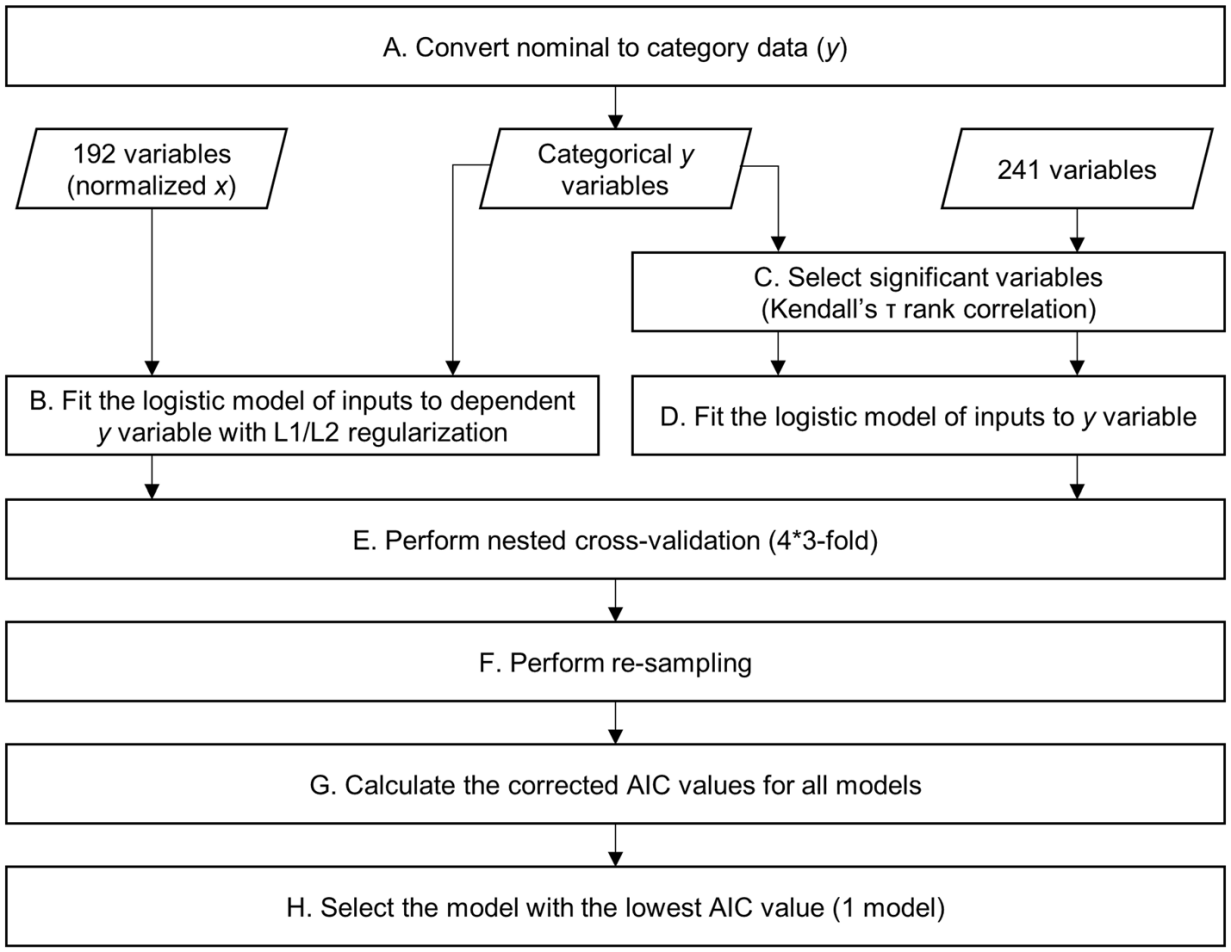
Estimation process starting with variable preprocessing (**A**), modeling with (**B**) and without (**C,D**) the regularization, nested cross-validation (**E**), re-sampling (**F**), model parameter calculation (**G**), and model selection (**H**).

Three smaller subsets, results of subset selection model, and the complete dataset (i.e., 195 subjects) were independently analyzed. Even though, the lesion characteristics (e.g., occurrence and sparsity) across regions have been controlled for each subset, the region-wise distributions were not normal (0.5–33.6% total subjects with lesions in a region). The lesion degree measure was set as the dependent variable (*y*); the non-imaging features were used as the independent variables (*x*_1,2,3*…*_). Due to non-normal lesion distributions, analyzing nominal data (in %) of the dependent variable was unlikely feasible. Therefore, the lesion degree data was converted to two categories (Step A in Fig. 5). The categories were indexed as 0 and 1 for lesion absence and presence, respectively.

To obtain straightforward explanations of useful tests, lesion estimation models were constructed based on the multivariate logistic regression (Eq. 2) for each AAL region.

2 $$P=\frac{1}{\left[1+{e}^{-\left({a}_{1}{x}_{1}+{a}_{2}{x}_{2}+\cdots +{a}_{n}{x}_{n}+b\right)}\right]}$$

where *x* is inputs, *a* is estimated coefficients, *b* is intercept, and *P* is probability of the presence/high lesion category. If *P* is lower than 0.5, subject data were estimated for the absence/low lesion category, and vice versa. The modeling was separately performed for regression analyses with and without the regularization process. The least absolute shrinkage and selection operator (LASSO) and Ridge regression methods implements L1 (*α* = 1) and L2 (*α* = 0) regularization, respectively. Those methods preferably use normalized variables as inputs because of penalized coefficients (Step B in Fig. 5). The normalization process was possibly applied on 192 variables (*x*_1,2…,192_; in 0–1 value range). On the other hand, for the analysis without the regularization process, inputs were selected (Step C in Fig. 5) among 241 independent variables (*x*_1,2…,241_) according to its significant relationships (Kendall’s *τ* rank correlation; *p* < 0.05) with the categorized and dependent variable (*y*). The selected inputs were then fitted to the logistic model (Step D in Fig. 5).

Estimation models were validated by 4*3 fold nested cross-validation (Step E in Fig. 5). The LASSO and Ridge methods optimized the tuning parameter (λ) during the nested cross-validation process. Meanwhile, the nested cross-validation for the regression analysis without the regularization was optimized by the highest validation sensitivity (i.e., true presence/high lesion category). The re-sampling process (100 times) was also performed to tune hyperparameter (Step F in Fig. 5). Four hundred models were obtained from the processes of nested cross-validation and re-sampling. However, those processes (Steps E and F) were unfeasibly executed for all regions because the number of data with presence/high lesion category was limited (< 4) in some regions. Only a single model was acquired in those regions. Akaike information criterion (AIC) values were computerized for all models (Step G in Fig. 5). The best model was selected among 400 models in accordance with the lowest AIC value (Step H in Fig. 5). All processes related to modeling and cross-validation declared in this sub-section (“Lesion modeling process”) were developed in the MATLAB environment (R2018b, The MathWorks, Inc.).

### Model implementation

**Figure 6:**
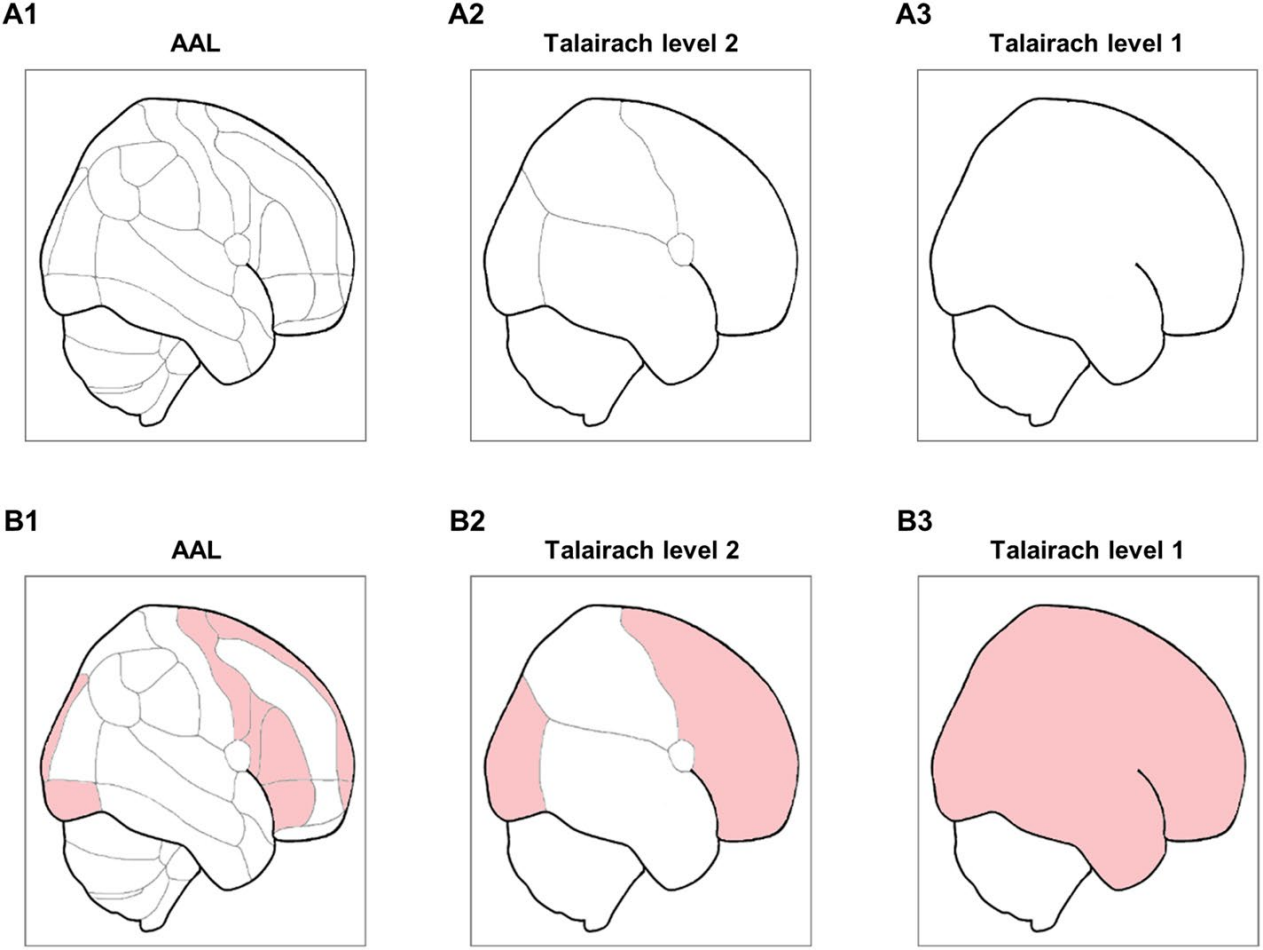
Spatial resolutions and the estimation of lesion occurrence in different resolutions. (**A**) Spatial registrations in AAL (A1), Talairach level 2 (A2), and Talairach level 1 (A3) atlases. (**B**) An example of lesion estimation in spatially modified resolutions [from AAL (B1) to Talairach levels 2 (B2) and 1 (B3)]. The red-colored regions denote regions with lesions.

Brain regions can be registered by different manners, such as the Talairach atlas. The Talairach levels 2 (i.e., lobes; 18 regions) and 1 (i.e., hemispheres; five regions) classify the brain with the smaller number of regions than the AAL registration does (Fig. 6A). For example, a single region of Talairach level 2 (e.g., left occipital lobe) comprises several AAL regions (e.g., left calcarine, left cuneus, left lingual, left superior-middle-inferior occipital gyrus). The spatially modified labeling, the transformation from AAL to Talairach labels, was done by the nearest neighbor method on the voxel-wise template [Statistical Parametric Mapping (SPM); www.fil.ion.ucl.ac.uk/spm/].

The selected models were applied on independent variables to estimate lesion categories for each AAL region. Three model parameters—accuracy (true absence/low and true presence/high), specificity (true absence/low), and sensitivity (true presence/high), were evaluated. Those parameters were also assessed as the same models of AAL regions were used to estimate lesions in spatially modified regions. If presence/high lesion categories (index 1) were estimated, the regions of Talairach levels 2 and 1 respected to AAL regions were also predicted having presence/high lesion categories (i.e., OR logic). Figure 6B shows an example of lesion category indexing for the spatially modified regions. Observing lesions in one or more AAL regions within a lobe (Talairach level 2) and a hemisphere (Talairach level 1) consequently brought an estimation of occurred lesion in the respected lobe (Fig. 6B2) and hemisphere (Fig. 6B3). To compare the effect of spatial modification in lesion estimation, the modeling process (see “Model for subset selection”) was directly carried out for each region of Talairach levels 2 and 1.

## Data Availability

The datasets used and/or analysed during the current study available from the corresponding author on reasonable request.
